# Phytate, iron, zinc, and calcium content of common Bolivian foods and their estimated mineral bioavailability

**DOI:** 10.1002/fsn3.1127

**Published:** 2019-08-02

**Authors:** Vanesa Castro‐Alba, Claudia Eliana Lazarte, Björn Bergenståhl, Yvonne Granfeldt

**Affiliations:** ^1^ Department of Food Technology, Engineering and Nutrition Lund University Lund Sweden; ^2^ Food and Natural Products Center San Simón University Cochabamba Bolivia

**Keywords:** calcium, estimated bioavailability, iron, phytate, plant‐based diet, zinc

## Abstract

There is a scarcity of information on mineral and phytate content in plant‐based foods in Bolivia. This study aimed to analyze iron, zinc, calcium, and phytate content and estimate the mineral bioavailability of foods consumed in Chapare, Bolivia. Minerals and phytate were analyzed, and bioavailability was estimated in 17 food samples. Leafy vegetables and green legumes had the highest mineral content, followed by pseudocereals. Estimated mineral bioavailability was low for cereals, dry legumes, pseudocereals, and flaxseeds foods mainly due to phytate content. But estimated zinc bioavailability for black cornmeal, yellow corn, and dry peas was moderate. Strong correlations (*p* < 0.01) were found between the three minerals, while phytate correlated negatively to iron, zinc, and calcium. To get an overview of the estimated mineral bioavailability of plant‐based diets, we have included foods, from the same area, analyzed in a previous study where the evaluated diet covers 80% of RNI for iron and zinc, but <40% of calcium. In conclusion, leafy vegetables and green legumes had the highest contents of minerals and the lowest phytate content of the foods analyzed in the study. The usage of processing strategies and dietary diversification to reduce phytate content would significantly improve estimated mineral bioavailability in plant‐based diets.

## 1 INTRODUCTION

Plant‐based products are the main staple foods for humans in many parts of the world, particularly in developing countries, although today there is an increasing interest in plant‐food diets in developed countries due to, among other reasons, an interest in improving the sustainable food systems. In a plant‐based diet, vegetables constitute an important source of all nutrients including minerals; however, they are also source of mineral inhibitors such as phytate, a main concern for the mineral bioavailability of foods (Gibson, Bailey, Gibbs, & Ferguson, 2010). According to the World Bank (2019) in Bolivia for year 2016, 16% children under 5 years suffer from malnutrition (low height for age). The National Institute of Statistics of Bolivia (INE, 2017) reported that 53.7% children under 5 years old suffer anemia, being 60% of them from rural areas, and the prevalence of anemia among women between 19 and 49 years old was 29.8%. According to the evaluation reported by Lazarte, Carlsson, Almgren, Sandberg, and Granfeldt (2015), the dietary pattern of inhabitants of Chapare, a tropical rural area in Cochabamba, Bolivia, is mainly based on plant foods such as tubers, cereals, and legumes with limited amounts of animal food sources including dairy products. This plant‐based diet, which is consumed in several rural areas of the country, might contain high levels of antinutrients and, therefore, be one of the main causes of micronutrient deficiency in Bolivia.

Phytate, *myo*‐inositol‐1,2,3,4,5,6,‐hexakisphosphate (IP6), is a naturally occurring compound in basic commodities like cereals, legumes, tubers, and oilseeds. IP6 is accumulated during the ripening period and represents between 60% and 90% of total phosphorous content in mature seed and grains (Reddy & Sathe, 2001). Phytate occurs when phytic acid, a negatively charged molecule, binds to mono‐ and divalent dietary mineral cations, forming very stable phytate complexes at neutral pH (Harland & Morris, 1995; Morris & Hill, 1996). Thus, the presence of phytate in the diet normally decreases the bioavailability of divalent cations such as iron, zinc, and calcium in the gastrointestinal tract, and pH of the small intestine (6–7) increases the dissociation and formation of phytate‐divalent cation complexes that precipitate, making them less available for absorption into the human body (Schlemmer, Frolich, Prieto, & Grases, 2009). The presence of active dietary phytase plays an important role in the hydrolysis of phytate, and its importance for mineral absorption is enhanced by the lack of endogenous phytase in the small intestine (Lestienne, Icard‐Verniere, Mouquet, Picq, & Treche, 2005; Lopez, Leenhardt, Coudray, & Remesy, 2002).

The absorption and availability of iron, zinc, and calcium in the human body can be predicted by calculating molar ratios of phytate to mineral (Lestienne et al., 2005; Lopez et al., 2002; Luo, Gu, Han, & Chen, 2009). There is evidence that a molar ratio of phytate to iron (Phy:Fe) above 1 has a negative effect on iron absorption; a preferable molar ratio is 0.4 (Hurrell & Egli, 2010; Magallanes‐Lopez et al., 2017). A phytate to zinc molar ratio (Phy:Zn) higher than 15 is associated with low estimated bioavailability, and Phy:Zn between 5 and 15 and below 5 is associated with moderate and high estimated bioavailability, with a zinc absorption corresponding to 15%, 30%, and 50%, respectively (Gibson & Ferguson, 1998; Lönnerdal, 2002; Magallanes‐Lopez et al., 2017). Calcium absorption can be impaired by a molar ratio of phytate to calcium (Phy:Ca) above 0.17 (Sandberg & Svanberg, 1991). High calcium content in food could exacerbate the negative effect of phytate on zinc absorption; a molar ratio of calcium to zinc of 100:1 has a synergetic effect on zinc, forming more stable calcium–zinc–phytate complexes at neutral pH. Therefore, a molar ratio of phytate·calcium to zinc (Phy·Ca:Zn) has been suggested as a better predictor of zinc absorption, and a molar ratio higher than 200 may have a negative effect on zinc bioavailability (Fordyce, Forbes, Robbins, & Erdman, 1987; Oberleas & Harland, 1981).

To date, there is a scarcity of information on mineral content and inhibitors of them in foods that make up typical diets in Latin America. Although these countries have national food composition tables, in most cases, information on the nutritional composition of foods has been taken from other databases such as the USDA Food Composition Databases, and Bolivia is no exception to this trend. To our knowledge, there is only one report about phytate content and its implication on iron, zinc, and calcium estimated bioavailability of some foods in the tropical rural area of Chapare (foods that are also commonly consumed across the whole country) (Lazarte, Carlsson, et al., 2015). More studies are required in order to determine mineral and phytate content, as well as their implications for the bioavailability of minerals, in plant‐based foods commonly consumed in Bolivia. Therefore, the present study aimed to determine the content of iron, zinc, calcium, and phytate and to estimate the bioavailability of these minerals by calculating their corresponding molar ratios in 17 foods commonly consumed in five villages in Chapare, Bolivia. The data generated in this study will be of importance to help elucidate the causes of mineral deficiencies (Fe, Zn) that have been found in these areas. The data provided in the present paper are also highly relevant for international databases such as FAO/INFOODS/IZiNCG Global Food Composition Database for Phytate (Dahdouh et al., 2019).

## 2 MATERIAL AND METHODS

### 2.1 Food samples

The food selection for the study included 17 food samples (Table 1); 10 food samples were selected from the food frequency questionnaire (FFQ) conducted in 2014 by Lazarte, Carlsson, et al. (2015). The FFQ was carried out among 65 participants in the villages of Eterazama and Villa Tunari located in the tropical area of Chapare, approximately 160 km northeast of Cochabamba, Bolivia. The FFQ included 72 food items from which the 16 most consumed foods were analyzed and reported in a previously published article (Lazarte, Carlsson, et al., 2015). In addition, barley flour and flaxseeds were included because they are common in rural areas of Bolivia, and they were registered in the dietary assessments conducted in Chapare (Lazarte, Soto, et al., 2015). Cassava and new cocoyam leaves were selected as these leaves are widely available in the region, and according to the literature, they have a high mineral content (Latif & Müller, 2015). Finally, besides quinoa, two other pseudocereals, amaranth and canihua, were included, as these food staples often feature in meals that are offered to children in schools. Twelve out of the 17 food samples were purchased in markets in five villages (Chimore, Eterazama, Ivirgarzama, Shinaota, and Villa Tunari) located in Chapare in March 2014. Two samples of 1 kg each were obtained from each village—in total 10 samples of each food. Fresh cassava and walusa leaves (two samples of 1 kg each) were picked from the fields in each village. The food samples were transported to the nutrition laboratory of the Center of Food and Natural Products, San Simón University (CAPN—UMSS), approximately 160 km from Chapare. The samples were then stored at room temperature or in the fridge at 4°C (leafy vegetables in plastic bags) until sample preparation (1–5 days). Three food samples (quinoa, amaranth, and canihua) were purchased in markets in Cochabamba city.

**Table 1:** Food samples obtained in five villages in Chapare, Bolivia, and preparation method

Food groups/Name	*N* a	Scientific name	Preparation method^b^
Prepared foods			
Cereals			
Barley flour	10	*Hordeum vulgare*	Ready‐to‐eat^c^
Black cornmeal	10	*Zea mays*	Soaked (4 hr) and boiled (20 min)^d^
Yellow corn	10	*Z. mays*	Soaked (12 hr) and boiled (80 min)
Oat	10	*Avena sativa*	Boiled (5 min)
Green legumes			
Green beans	10	*Phaseolus vulgaris*	Chopped (~2 cm^2^) and boiled (10 min)
Green peas	10	*Pisum sativum*	Seed removed manually from pods and boiled (10 min)
Dry legumes			
Dry fava beans	10	*Vicia faba*	Soaked (12 hr), manually dehulled after soaking and boiled (20 min)
Kidney beans	10	*P. vulgaris*	Soaked (12 hr) and boiled (60 min)
Dry peas	10	*P. sativum*	Soaked (12 hr) and boiled (20 min)
Peanuts	10	*Arachis hypogaea*	Soaked (2 hr) and boiled (60 min). Defatted for phytate analysis
Leafy vegetables			
Cassava leaves	10	*Manihot esculenta*	Chopped (2 cm) and boiled (10 min)
New cocoyam leaves	10	*Xanthosoma sagittifolium*	Chopped (2 cm) and boiled (5 min)
Other			
Sweet potato	10	*Ipomea batatas*	Peeled, cut into pieces (~2 cm^2^) and boiled (25 min)
Flaxseeds	10	*Linum usitatissimum*	Boiled (5 min); defatted for phytate analysis
Raw foods			
Pseudocereals			
Quinoa	10	*Chenopodium quinoa*	Raw grains
Canihua	10	*Chenopodium pallidicaule*	Raw grains
Amaranth	10	*Amaranthus caudatus*	Raw grains

a Five food samples duplicate.

b Soaking process was carried out at room temperature ~ 25°C; boiling temperature was ~92°C. Distilled water was used for soaking and cooking, and water was discarded afterward, except for black cornmeal, oat and flaxseeds where water was kept (porridge). All prepared samples were dried at 60°C for 24 hr.

c The preparation of ready‐to‐eat barley includes toasting and milling of barley seeds.

d The cornmeal preparation includes dehulling corn seeds either with wood ashes or with calcium carbonate.

Among the 17 foods included, only cassava and new cocoyam leaves are cultivated in the tropical rainforest of Chapare. Quinoa and canihua are cultivated in the highlands with an average temperature between 5 and 10°C and altitude above 3,000 m above sea level (m.a.s.l.). Black and yellow corn, barley, oats, green beans, green peas, dry fava beans, dry peas, peanuts, and amaranth are mainly produced in the valley at around 2,000–3,000 m.a.s.l., at a milder temperature (15–25°C) than in the highlands. Kidney beans and flaxseeds are cultivated in the lowlands of Bolivia where the temperature is higher, between 22 and 25°C, and the altitude is 200–800 m.a.s.l. (Hudson & Hanratty, 1991; UNISDR, 2012).

### 2.2 Sample preparation

Sample preparation was conducted for each food sample following common preparation methods used in the study area (i.e., soaking, boiling, cooking until tissue was soft), except for pseudocereals, which were analyzed without further preparation. For procedures such as soaking and cooking, distilled water was used in all cases and discarded afterward, except for black cornmeal, oats, and flaxseed, where the boiling water was kept (i.e., to make porridge). Table 1 provides details on sample preparation for each food item. After sample preparation, a portion of 100 g of each food was dried at 60°C for 24 hr (heating oven model ED23 Binder) and stored at 4°C until analysis.

### 2.3 Moisture analysis

The moisture content was determined by measuring water loss after drying. Five grams (±0.0001 g) of each sample was weighed and dried at 105°C (heating oven model ED23 Binder) until a constant weight was obtained (AOAC, 2000).

### 2.4 Mineral content analysis

Mineral content was determined following the method described by Lazarte, Carlsson, et al. (2015). Duplicate samples of 0.5 g (±0.0001 g) were placed in Teflon vessels, and 5 ml of nitric acid (65% V/V; Merck) and 2 ml of hydrogen peroxide (30% V/V; Merck) were added. The samples were acid digested in a microwave reaction system (Model Multiwave PRO; Anton Paar CO.) for 1 hr. After digestion, the samples were diluted to 25 ml with deionized water. Iron, zinc, and calcium were analyzed by flame atomic absorption with air‐acetylene flame (Model AAnalyst 200; PerkinElmer Corp.) at 248.3, 213.9, and 422.7 nm wavelength, respectively. For calcium analysis, lanthanum oxide (1% w/V) was added to standards and samples before analysis to prevent phosphorous interference. To avoid any mineral contamination, all materials and glassware were washed with a solution of nitric acid 3% and double‐rinsed with distilled water and deionized water.

A calibration curve of five points was prepared for each mineral (40–4,000 μg/L) from certified Atomic Absorption Standard Solutions (Iron and Zinc; Pure Standards for atomic absorption PerkinElmer Corp., Calcium Sigma‐Aldrich Co). Quality control of each microwave digestion (16 Teflon vessels) was performed by placing a certified reference material (rice flour, IRMM 804 FLUKA; Sigma‐Aldrich Co.) in one vessel. In another vessel, a blank was prepared with nitric acid and hydrogen peroxide without addition of food material. Each digestion round was composed of a vessel with reference material, a blank vessel and 14 vessels containing food samples. The mineral content of the reference material and blank was determined by atomic absorption spectrophotometry at the same time as the food samples to check the precision and accuracy of the procedure. One standard (1,000 μg/L) from the standard curve was controlled after each 10 measurements to ensure the stability of the instrument; if needed, the calibration was repeated. The relative standard deviation (% RSD) was below 5% for each measurement.

### 2.5 Phytate content analysis

Phytate content, as *myo*‐inositol hexakisphosphate, was analyzed by high‐performance ion chromatography (HPIC) according to the method described by Carlsson, Bergman, Skoglund, Hasselblad, and Sandberg (2001) and Lazarte, Carlsson, et al. (2015) with slight modifications: Duplicate samples of 0.5 g were extracted with 20 ml of 0.5 M HCl for 2 hr at room temperature under constant stirring. Extracts were centrifuged (Model Allegra^®^ X‐15r; Beckman Coulter) at  2,851 g for 10 min at 20°C. Supernatant was recovered and frozen overnight, thawed, and centrifuged (Model Optima™ LE‐80k; Beckman Coulter) at 12,348 g for 10 min at 20°C. An aliquot of 2 ml of supernatant was filtered through a 0.2‐μm syringe filter disk, and 50 μl of supernatant was injected and analyzed by HPIC with CarboPac PA‐100 (4 × 250 mm) analytical column and a CarboPac PA‐100 (4 × 50 mm) guard column (Dionex Corp.). Detection and quantification of phytate were made after a postcolumn reaction with Fe(NO_3_).9H_2_O (99.99% trace metal basis; Aldrich) in 2% HClO_4_; the absorbance was monitored at 290 nm in a UV detector (HP Agilent series 1050). Phytate dodecasodium salt hydrate (Sigma‐Aldrich) was used as standard. The limit of detection of the method was 60 mg/100 g.

### 2.6 Estimation of mineral relative bioavailability

The relative bioavailability of iron, zinc, and calcium was estimated by the molar ratios of phytate to mineral, that is, Phy:Fe, Phy:Zn, Phy:Ca, and Phy·Ca:Zn. The molar ratios were calculated using 660.3 g/mol as the molecular weight of phytate.

### 2.7 Statistical analysis

The results are presented as means and standard deviation, which were calculated using Microsoft Excel 2010. In order to estimate which mineral is the most affected by the phytate content, mineral content and phytate, as well as phytate and molar ratios, were correlated by Pearson's bivariate correlation test using SPSS Statistics 24 (SPSS Inc., IBM Corporation, Armok, USA). Principal component analysis (PCA), which includes 33 common food components (16 analyzed in a previous study and 17 samples presented in this study) of the Chapare diet, was performed to find out the relationship among phytate content, minerals (iron, zinc, and calcium), and food groups, using The Unscrambler^®^ X 10.2 (CAMO software AS). All the data were normalized for this analysis.

## 3 RESULTS

### 3.1 Mineral and phytate content

The results of mineral and phytate content in dry matter, and the moisture percentage of cereals, pseudocereals, green and dry legumes, leafy vegetables, tubers, and flaxseeds are summarized in Table 2. Among the studied foods, leafy vegetables and green legumes had high contents of iron (from 7.95 mg/100 g in green peas to 17.2 mg/100 g in cassava leaves). Iron contents in flaxseeds and pseudocereals, but quinoa (which was lower), were comparable to green legumes. Iron contents in cereals and dry legumes were similar, except for peanut that was lower. The lowest content of iron was found in sweet potato. Regarding zinc, leafy vegetables also had high content of these mineral. Flaxseeds and green legumes had comparable concentrations of zinc. Dry legumes, pseudocereals, and cereals (but yellow corn and barley flour, which were lower) had similar content of zinc. Zinc concentration was the lowest in sweet potato. The highest content of calcium was found in leafy vegetables, flaxseeds, and green legumes, except green peas. Dry legumes, pseudocereals, sweet potato, and cereals, but yellow corn and barley flour, had similar contents of calcium. The phytate content among different food groups varied greatly. Oat in cereals, dry fava beans in dry legumes, and amaranth in pseudocereals have higher content of phytate rather that the rest of the foods included in the different groups. The lowest phytate content, below the limit of detection of the method (60 mg/100 g), was found in green beans, peas, leafy vegetables, and sweet potato.

**Table 2:** Mineral and phytate content in prepared foods regularly consumed in Chapare, Bolivia, and raw pseudocereals, mean ± *SD* (min to max) per 100 g dry matter (DM)

Food groups/Name	Moisture g/100g DM	Iron mg/100 g DM	Zinc mg/100 g DM	Calcium mg/100 g DM	Phytate mg/100 g DM
Cereals					
Barley flour	6.67 ± 0.78	8.13 ± 2.1 (5.14–11.6)	1.16 ± 0.72 (0.71–2.76)	23.2 ± 2.8 (17.7–27.7)	230 ± 26 (189–274)
Black cornmeal	84.4 ± 1.1	9.01 ± 1.7 (5.91–10.6)	5.19 ± 0.88 (3.87–6.61)	54.7 ± 33^a^ (15.7–119)	559 ± 131 (401–760)
Yellow corn	52.0 ± 1.5	3.18 ± 0.99 (2.06–5.24)	3.72 ± 0.66 (2.84–4.73)	5.00 ± 4.4 (1.11–14.1)	526 ± 121 (372–727)
Oat	84.6 ± 5.9	4.55 ± 0.49 (3.67–5.18)	3.17 ± 0.39 (2.50–3.68)	40.6 ± 7.7 (32.4–53.5)	2,618 ± 295 (2,204–2,993)
Green legumes					
Green beans	93.3 ± 0.84	11.4 ± 1.7 (9.04–14.6)	5.12 ± 0.63 (4.30–6.05)	242 ± 11 (223–259)	<60
Green peas	75.2 ± 4.6	7.95 ± 1.4 (5.56–9.83)	5.86 ± 2.3 (1.49–7.55)	58.0 ± 11 (34.7–73.6)	<60
Dry legumes					
Dry fava beans	66.7 ± 1.4	6.25 ± 0.83 (4.92–7.46)	4.45 ± 0.31 (3.93–4.83)	36.5 ± 8.3 (22.6–47.0)	2,248 ± 671 (1,571 ± 3,648)
Kidney beans	62.9 ± 2.4	7.74 ± 0.85 (6.53–8.97)	4.10 ± 0.45 (3.29–4.67)	65.7 ± 10 (46.6–83.2)	719 ± 118 (501–932)
Dry peas	57.0 ± 4.0	5.49 ± 0.58 (4.55–6.30)	3.73 ± 0.74 (2.90–4.57)	42.9 ± 3.5 (38.1–47.9)	344 ± 78 (173–444)
Peanuts	45.1 ± 4.0	2.12 ± 0.20 (1.92–2.50)	4.05 ± 0.27 (3.72–4.46)	66.2 ± 12.2 (39.9–81.9)	832 ± 91 (749–992)
Leafy vegetables					
Cassava leaves	82.2 ± 1.9	17.2 ± 4.1 (11.8–23.3)	12.2 ± 0.85 (11.4–13.7)	264 ± 69 (178–391)	<60
New cocoyam leaves	91.9 ± 0.83	10.4 ± 0.92 (8.82–11.8)	8.07 ± 3.9 (3.18–15.8)	650 ± 23 (605–682)	<60
Others					
Sweet potato	72.4 ± 3.9	0.92 ± 0.35 (0.59–1.59)	0.63 ± 0.12 (0.43–0.86)	45.4 ± 13 (30.4–68.7)	<60
Flaxseeds	73.6 ± 2.0	8.20 ± 1.4 (6.46–10.0)	6.31 ± 0.62 (5.37–7.53)	192 ± 17 (154–211)	997 ± 141 (787–1,232)
Pseudocereals					
Quinoa	9.43 ± 0.11	4.96 ± 0.36 (4.40–5.46)	3.36 ± 0.36 (3.06–3.92)	58.8 ± 1.5 (56.4–60.6)	844 ± 51 (752–926)
Canihua	10.6 ± 0.17	12.2 ± 1.2 (11.3–14.4)	4.07 ± 0.39 (3.69–4.56)	71.7 ± 1.7 (70.8–75.0)	796 ± 111 (688–988)
Amaranth	10.4 ± 0.10	7.97 ± 0.55 (7.02–8.80)	3.70 ± 0.30 (3.43 – 4.18)	66.3 ± 6.3 (60.9–77.3)	1,382 ± 156 (1,124–1,571)

Note

Limit of detection 60 mg/100 g.

a Calcium carbonate may have been used during preparation of cornmeal, see Table 1.

### 3.2 Estimated mineral bioavailability

The calculated molar ratios of Phy:Fe, Phy:Zn, Phy:Ca, and Phy·Ca:Zn for estimation of mineral bioavailability are shown in Table 3. All the studied food groups had a Phy:Fe molar ratio above the threshold that estimates a good iron bioavailability. Only black cornmeal and yellow corn in cereals, and dry peas in dry legumes had Phy:Zn and Phy·Ca:Zn molar ratios below the critical values of 15 and 200, respectively, meaning that the estimated zinc bioavailability in these foods is moderate. The Phy:Ca molar ratio was above the threshold of 0.17 for all the studied foods; therefore, the estimated calcium bioavailability is low.

**Table 3:** Phy:mineral molar ratio of prepared foods commonly consumed in Chapare, Bolivia, and raw pseudocereals, mean ± *SD* (min to max) in dry matter

Food groups/Name	Phy:Fe	Phy:Zn	Phy:Ca	Phy·Ca:Zn
Cereals				
Barley flour	2.57 ± 0.79 (1.54–3.81)	24.0 ± 8.5 (9.43–33.1)	0.61 ± 0.11 (0.41–0.74)	140 ± 56 (50.6–209)
Black cornmeal	5.34 ± 1.2 (3.77–7.38)	10.9 ± 2.2 (8.68–15.2)	0.89 ± 0.45 (0.34–1.56)	181 ± 133 (37.3–393)
Yellow corn	15.1 ± 5.9 (8.90–27.8)	14.6 ± 4.3 (9.77–23.7)	12.2 ± 11 (1.65–37.0)	18.4 ± 14 (5.92–44.3)
Oat	49.2 ± 6.9 (37.8–57.0)	82.4 ± 9.8 (68.5–96.4)	4.00 ± 0.60 (3.24–5.06)	820 ± 199 (623–1,178)
Green legumes				
Green beans	<0.46 (<0.35–0.56)	<1.43 (<0.98–1.38)	<0.02 (<0.01–0.02)	<71.2 (<55.5–86.5)
Green peas	<0.66 (<0.52–0.91)	<0.87 (<0.79–1.06)	<0.07 (<0.05–0.10)	<15.2 (<10.0–28.7)
Dry legumes				
Dry fava beans	30.2 ± 6.2 (21.8–42.4)	46.2 ± 10 (33.3–62.5)	3.94 ± 1.5 (2.36–6.90)	456 ± 166 (271–724)
Kidney beans	7.97 ± 1.8 (5.95–12.1)	17.4 ± 2.7 (14.4–23.0)	0.67 ± 0.12 (0.54–0.92)	287 ± 71 (175–410)
Dry peas	5.33 ± 1.3 (2.83–7.24)	9.30 ± 2.4 (5.58–12.5)	0.49 ± 0.11 (0.27–0.61)	99.2 ± 24 (55–129)
Peanuts	33.5 ± 5.5 (25.8–43.7)	20.4 ± 2.8 (17.5–26.4)	0.79 ± 0.19 (0.59–1.21)	336 ± 71 (206–437)
Leafy vegetables				
Cassava leaves	<0.31 (<0.22–0.43)	<0.49 (<0.43–0.52)	<0.01 (<0.01–0.02)	<34.3 (<20.4–60.8)
New cocoyam leaves	<0.47 (<0.22–0.58)	<0.91 (<0.38–1.87)	<0.01 (<0.01–0.01)	<147 (<64.1–313)
Others				
Sweet potato	<6.14 (<3.20–8.66)	<9.81 (<6.93–13.9)	<0.09 (<0.05–0.12)	<109 (<72.1–158)
Flaxseeds	10.5 ± 2.1 (7.30–14.3)	15.8 ± 2.3 (12.1–18.1)	0.32 ± 0.06 (0.23–0.41)	748 ± 105 (622–871)
Pseudocereals				
Quinoa	14.5 ± 1.5 (11.7–17.3)	25.0 ± 2.1 (21.3–27.6)	0.67 ± 0.08 (0.59–0.80)	367 ± 36 (308–415)
Canihua	5.6 ± 1.0 (4.1–7.0)	19.4 ± 2.0 (15.3–22.3)	0.67 ± 0.08 (0.59–0.80)	348 ± 41 (270–401)
Amaranth	14.8 ± 2.5 (11.7–17.6)	37.5 ± 6.7 (29.3–44.9)	1.27 ± 0.07 (1.12–1.36)	628 ± 165 (446–865)

The relationship between the content of phytate and the respective minerals was negative (zinc *p* < 0.05, iron and calcium *p* < 0.01) when all 17 food samples were included (Table 4). For the different food groups, the significant correlation was more random. There were negative correlations between phytate and iron (*p* < 0.05) in cereals, and between phytate and calcium (*p* < 0.05) in dry legumes. The correlation between phytate and zinc (*p* < 0.05) was positive for dry legumes. The correlations between zinc, iron, and calcium, respectively, were positive (*p* < 0.01) for all food samples; for the different groups, only pseudocereals showed the same result.

**Table 4:** Correlation coefficients for phytate, iron, zinc, and calcium content in 17 foods consumed in five villages in Chapare, Bolivia

Food groups/Name	Phytate	Iron	Zinc
Cereals			
Iron	−0.36^a^		
Zinc	0.09	0.14	
Calcium	0.28	0.53^b^	0.36^a^
Pseudocereals			
Iron	−0.18		
Zinc	−0.06	0.62^b^	
Calcium	0.18	0.74^b^	0.43^a^
Green legumes			
Iron	.^c^		
Zinc	.^c^	0.10	
Calcium	.^c^	0.74^b^	−0.18
Dry legumes			
Iron	0.18		
Zinc	0.40^a^	0.19	
Calcium	−0.38^a^	−0.19	0.06
Leafy vegetables			
Iron	.^c^		
Zinc	.^c^	0.52^a^	
Calcium	.^c^	−0.80^b^	−0.58^b^
All food samples			
Iron	−0.24^b^		
Zinc	−0.19^a^	0.66^b^	
Calcium	−0.32^b^	0.47^b^	0.58^b^

a Correlation is significant at the 0.05 level (two‐tailed).

b Correlation is significant at the 0.01 level (two‐tailed).

c Cannot be computed because at least one of the variables is constant.

## 4 DISCUSSION

Given the relevance of plant‐based foods in the diets of rural populations in developing countries, it is important to know the content of essential minerals in these foods, along with the content of inhibitors that could affect mineral bioavailability. The results of the present study describing mineral and phytate content in prepared, regularly consumed food in Cochabamba, Bolivia, are highly relevant for developing countries in general, where diets are mainly composed of plant‐based foods. In this type of diet, mineral bioavailability can be significantly affected by phytate content, which may in turn lead to mineral deficiencies.

Leafy vegetables (cassava and new cocoyam leaves) are significant in that among the food groups tested, they have the highest content of iron, zinc, and calcium and low phytate content (below the limit of detection). The mineral content of cassava leaves is comparable to values reported from Brazil, Nigeria, and Rwanda (Fe 12–22 mg/100 g DM, Zn 3.6–12 mg/100 g DM, Ca 737–1,630 mg/100 g DM) (Achidi, Ajayi, Maziya‐Dixon, & Bokanga, 2008; Chavez et al., 2000; Umuhozariho, Shayo, Msuya, & Sallah, 2014; Wobeto, Corrêa, Abreu, Santos, & Abreu, 2006) while that of new cocoyam leaves is comparable to values reported from Colombia (Fe 16–52 mg/100 g DM, Zn 8.1 mg/100 g DM, Ca 1,970–2,620 mg/100 g DM) (Leterme, Londono, Estrada, Souffrant, & Buldgen, 2005). The leaves collected, for this study, in the five villages in Chapare had a high mineral content but with a wide variation in iron and calcium content in cassava leaves, and in zinc content in new cocoyam leaves (Table 2). Wobeto et al. (2006) previously reported that the wide variation in nutritional content, including minerals in cassava leaves from Brazil, depends on differences in cultivars, plant age, and chemical composition of the soil. The high mineral content and low phytate levels in leafy vegetables suggest that the bioavailability of the studied minerals might not be affected by the presence of phytate. However, the bioavailability of iron and zinc might be impaired by polyphenols content and calcium bioavailability could be negatively affected by the oxalate content in cassava leaves (Latif & Müller, 2015). Comparing the leaves with their corresponding roots, our findings show that cassava and new cocoyam leaves are more concentrated in iron, zinc, and calcium and lower in phytate content than previously published values from each root purchased from the same area analyzed (Lazarte, Carlsson, et al., 2015). In many countries in Africa and some in Asia, cassava leaves are a regular part of diets in rural areas as they are available throughout the year. From a nutrition point of view, they are important for their high content of proteins and micronutrients (Latif & Müller, 2015). In Bolivia, the most commonly consumed part of cassava and new cocoyam is the root. However, from informal interviews with inhabitants of Chapare, it transpired that both types of leaves are used for cooking. The cooked leaves are typically served as a sauce combined with other vegetables and eaten with rice, potatoes, or cassava roots (Lazarte, Soto, et al., 2015). Therefore, dietary diversification by the inclusion of leafy vegetables in diets might enhance their nutrient profile; however, further studies are necessary to determine the effect of polyphenols and oxalates on mineral bioavailability of these foods.

Green legumes (green beans and green peas) are also rich in minerals that are required for the normal growth and development of seeds. Regarding green beans, our findings were somewhat higher for iron (9.04–14.6 mg/100 g DM) and zinc (4.30–6.05 mg/100 g DM) but lower for calcium (223–259 mg/100 g DM) than reported values from Spain (Fe 5.5–7.1 mg/100 g DM, Zn 4.1–5.0 mg/100 g DM, Ca 575–710 mg/100 g DM) (Martínez et al., 1998). Concerning green peas, our results show similar iron contents, somewhat higher zinc contents, and lower calcium contents than reported values from other Spanish and Czech studies (Fe 5.77–9.2 mg/100 g DM, Zn 2.6–4.8 mg/100 g DM, Ca 84–133 mg/100 g DM) (Koplík et al., 2004; Periago et al., 1996). The low levels of phytate in immature green legumes (<60 mg/100 g DM) compared to matured and dry legumes (344–2,248 mg/100 g DM) are notable due to the accumulation pattern of inorganic phosphorous as phytate, which increases with seed development with the highest content reached at seed maturity (Koplík et al., 2004; Reddy & Sathe, 2001). As in the case of leafy vegetables, the estimated bioavailability of minerals for green legumes is also high (Phy:Fe 0.46–0.66, Phy:Zn 0.87–1.4, Phy:Ca 0.02–0.07, Phy·Ca:Zn 15–71).

Dry legumes are an important source of minerals according to the Food and Agricultural Association (FAO) and are regularly consumed in tropical rural areas of Bolivia. Lazarte, Carlsson, et al. (2015) reported that dry fava beans and peanuts are consumed at least once or twice per month by 62% and 84% of the participants in Chapare, respectively. Dry peas and kidney beans are consumed with the same frequency by 91% of participants. For dry legumes, the mineral and phytate contents are generally higher than in cereals and tubers. In fava beans, our findings (Table 2) for iron are higher, and lower for zinc and calcium than the values reported for cooked fava beans from Egypt (Fe 6.1 mg/100 g DM, Zn 11 mg/100 g DM, 208 mg/100 g DM) (Khalil & Mansour, 1995). In kidney beans, the values for iron, zinc, and calcium were somewhat similar to those reported for red kidney beans from Canada (Fe 5.37 mg/100 DM, Zn 3.0 mg/100 g DM, Ca 78 mg/100 g DM) (Wang, Hatcher, Tyler, Toews, & Gawalko, 2010). In dry peas, the iron and zinc contents were comparable to and calcium was lower than the reported values from six varieties of soaked and cooked peas from Canada (Fe 4.09–5.11 mg/100 g DM, Zn 2.18–3.16 mg/100 g DM, Ca 81.6–108.8 mg/100 g DM) (Wang, Hatcher, & Gawalko, 2008). In peanuts, iron content was similar to and zinc and calcium content were higher than values reported from Benin and Bolivia (Fe 2.4–2.54 mg/100 g DM, Zn 3.0–3.3 mg/100 g DM, Ca 46–50 mg/100 g DM) (Lazarte, Carlsson, et al., 2015; Mitchikpe et al., 2008). Regarding phytate, our values are comparable with those reported for dry fava beans from Spain, 1,460–2,380 mg/100 g DM (Alonso, Aguirre, & Marzo, 2000); kidney beans from Turkey, 611–655 mg/100 g DM (Nergiz & Gökgöz, 2007); dried peas from Spain and Canada, 346–850 mg/100 g DM (Abd‐El‐Hady & Habiba, 2003; Periago et al., 1996; Wang et al., 2008); and peanuts from Benin and Bolivia, 483–2,071 mg/100 g DM (Lazarte, Carlsson, et al., 2015; Mitchikpe et al., 2008). The molar ratios of iron (Phy:Fe 5.33–33.5), zinc (Phy:Zn 17.4–46.2), and calcium (Phy:Ca 0.49–3.94) for dry legumes were above the critical values, which means that the estimated bioavailability of these minerals is low and is mainly affected by the phytate content. Dry peas had a moderate estimated bioavailability of zinc (Phy:Zn 9.30, Phy·Ca:Zn 99.2); this may be due to low phytate content rather than the content of zinc and the effect of soaking prior to boiling on this food sample. During the soaking process, the activation of endogenous dietary enzyme, phytase, may occur, resulting in lower phytate content; also, minerals may leak and dissolve into the soaking water (Reddy & Sathe, 2001).

Pseudocereals are a healthy alternative to conventional cereals and high in a wide range of nutrients (Haros & Schönlechner, 2017; Repo‐Carrasco‐Valencia, Encina, Binaghi, Greco, & Ronayne de Ferrer, 2010). Pseudocereals (in this study quinoa, canihua, and amaranth) have a higher content of iron, zinc, and calcium than conventional cereals, comparable to dry legumes. Similar contents of iron (4.96 mg/100 g DM) and zinc (3.36 mg/100 g DM) were previously reported for quinoa from Bolivia (Fe 5.4 mg/100 g DM, Zn 3.6 mg/100 g DM). For calcium (58.8 mg/100 g DM), threefold higher contents were reported (Ca 176 mg/100 g DM) (Lazarte, Carlsson, et al., 2015). The calcium content, which is located in the pericarp and seed coat, may be more or less affected by the abrasion process for elimination of saponins, which are found mainly in the outer layer of the seed (Konishi, Hirano, Tsuboi, & Wada, 2004; Ruales & Nair, 1993). Lower content of minerals has previously been reported for canihua (Fe 2.5 mg/100 g DM, Zn 2.8 mg/100 g DM, Ca 66 mg/100 g DM), and for amaranth seeds from Peru (Fe 5.0 mg/100 g DM, Zn 1.2 mg/100 g DM, Ca 28 mg/100 g DM) (Repo‐Carrasco‐Valencia et al., 2010; Villa, Russo, Kerbab, Landi, & Rastrelli, 2014). The phytate content in pseudocereals is moderate to high in this study and in agreement with the values previously reported for quinoa (752–2,470 mg/100 g DM), canihua (800 mg/100 g DM), and amaranth (1,393 mg/100 g DM) (Lazarte, Carlsson, et al., 2015; Repo‐Carrasco‐Valencia, Acevedo de La Cruz, Icochea Alvarez, & Kallio, 2009; Sanz‐Penella, Wronkowska, Soral‐Smietana, & Haros, 2013; Valencia, Svanberg, Sandberg, & Ruales, 1999). Although pseudocereals have relatively high mineral content, the estimated availability (Phy:Fe 5.6–15, Phy:Zn 19–36, Phy:Ca 0.67–1.3, Phy·Ca:Zn 348–628) is low because of phytate content.

Cereals are the most consumed foods in tropical rural areas of Bolivia, representing 45% of dietary energy supply for the entire country (FAO, 2014). The mineral and phytate content in the most consumed cereals in Bolivia have previously been reported (Lazarte, Carlsson, et al., 2015) and are now complemented with the results of the present study on other common cereal products. Black corn prepared as a meal has a mineral content similar to pseudocereals. Traditionally, black cornmeal preparation involves the separation of pericarp and tip caps from kernels using either wood ashes or calcium carbonate and water. This procedure, called lime‐cooking, may explain the high content of calcium and higher values of iron and zinc than in whole corn kernels and other cereals (FAO, 1992). To our knowledge, this is the first report on phytate content in black cornmeal (559 mg/100 g DM). The phytate content in yellow corn (526 mg/100 g DM) is lower than the range of reported value from Hungary, 800–1,020 mg/100 g DM (Hídvégi & Lásztity, 2002). Oats had the highest phytate content (2,204–2,993 mg/100 g DM) of all food samples analyzed, and from two to four times higher compared with previously reported values for oats produced in Sweden, 726–1,138 mg/100 g DM (Larsson & Sandberg, 1992; Sandberg & Svanberg, 1991). The barley flour had similar values to those reported from Sweden and Spain, 258–397 mg/100 g DM (Fredlund, Asp, Larsson, Marklinder, & Sandberg, 1997; Frontela, García‐Alonso, Ros, & Martínez, 2008). The estimated bioavailability of iron (Phy:Fe 2.57–49.2) and calcium (Phy:Ca 0.61–12.2) in cereals was low due to the molar ratios being higher than the critical values. Regarding zinc, estimated bioavailability was low for barley flour and oats (Phy:Zn 24.0 and 82.3, respectively), and moderate for black cornmeal and yellow corn (Phy:Zn 10.9 and 14.6, respectively).

Flaxseeds are a valuable source of omega‐3 fatty acids, fiber, and some essential minerals (Callegaro et al., 2011). In flaxseeds, the content of iron (6.46–10.0 mg/100 g DM) and zinc (5.37–7.53 mg/100 g DM) was higher than the values reported for six varieties of flaxseeds from Pakistan (Fe 4.45–7.12 mg/100 g DM, Zn 1.17–1.65 mg/100 g DM) (Khan, Sharif, Sarwar, Sameea, & Ameen, 2010). Calcium content (154–211 mg/100 g DM) was lower than the values reported from Brazil and Pakistan (310–460 mg/100 g DM) (Callegaro et al., 2011; Khan et al., 2010). Phytate content (997 mg/100 g DM) was lower than the reported values from Canada (1,597 mg/100 g DM) (Ratnayake et al., 1992). The estimated bioavailability of iron (Phy:Fe 10.5), zinc (Phy:Zn 15.8), and calcium (Phy:Ca 0.32) in the flaxseeds we tested may therefore be inhibited due to high phytate content.

Sweet potato is rich in starch and other nutrients (Desse, 2016), and the frequency of consumption of these tubers is between once and twice per month in Chapare (Lazarte, Carlsson, et al., 2015). Iron content (0.59–1.59 mg/100 g DM) was lower than the values (2.0 mg/100 g DM) reported for boiled sweet potato from Ethiopia (Umeta, West, & Fufa, 2005). Zinc (0.43–0.86 mg/100 g DM) and calcium content (30.4–68.7 mg/100 g DM) were similar to zinc (0.86 mg/100 g DM) and calcium content (68 mg/100 g DM) reported in the same study. We found low phytate content (below detection limit 60 mg/100 g) in these tubers while Umeta et al. (2005) reported 100 mg/100 g DM in boiled sweet potato.

The positive association (Table 4) found between minerals for the 17 foods analyzed in the present study may indicate that any strategy used to improve the dietary intake of one mineral might also have a positive effect on the others. However, this positive association is not valid for all food groups individually. Negative correlations or no associations for iron, zinc, and calcium with phytate were found for almost all food groups in the present study (Table 4). These results might be explained by the fact that minerals and phytate are not always found in the same part of the seeds or grains and that phytate can be linked to other elements such as magnesium or potassium or the variability of phytate depending of the maturity of the seeds or grains (Reddy & Sathe, 2001). Lee, Loh, Bong, Sarbini, and Yiu (2015) reported no correlation between phytate and minerals in a study carried out with 30 samples of two rice cultivars from Malaysia. Masum Akond, Crawford, Berthold, Talukder, and Hossain (2011) reported a negative correlation for phytate and iron, and calcium, and between phytate and zinc in 29 common bean genotypes.

**Figure 1:**
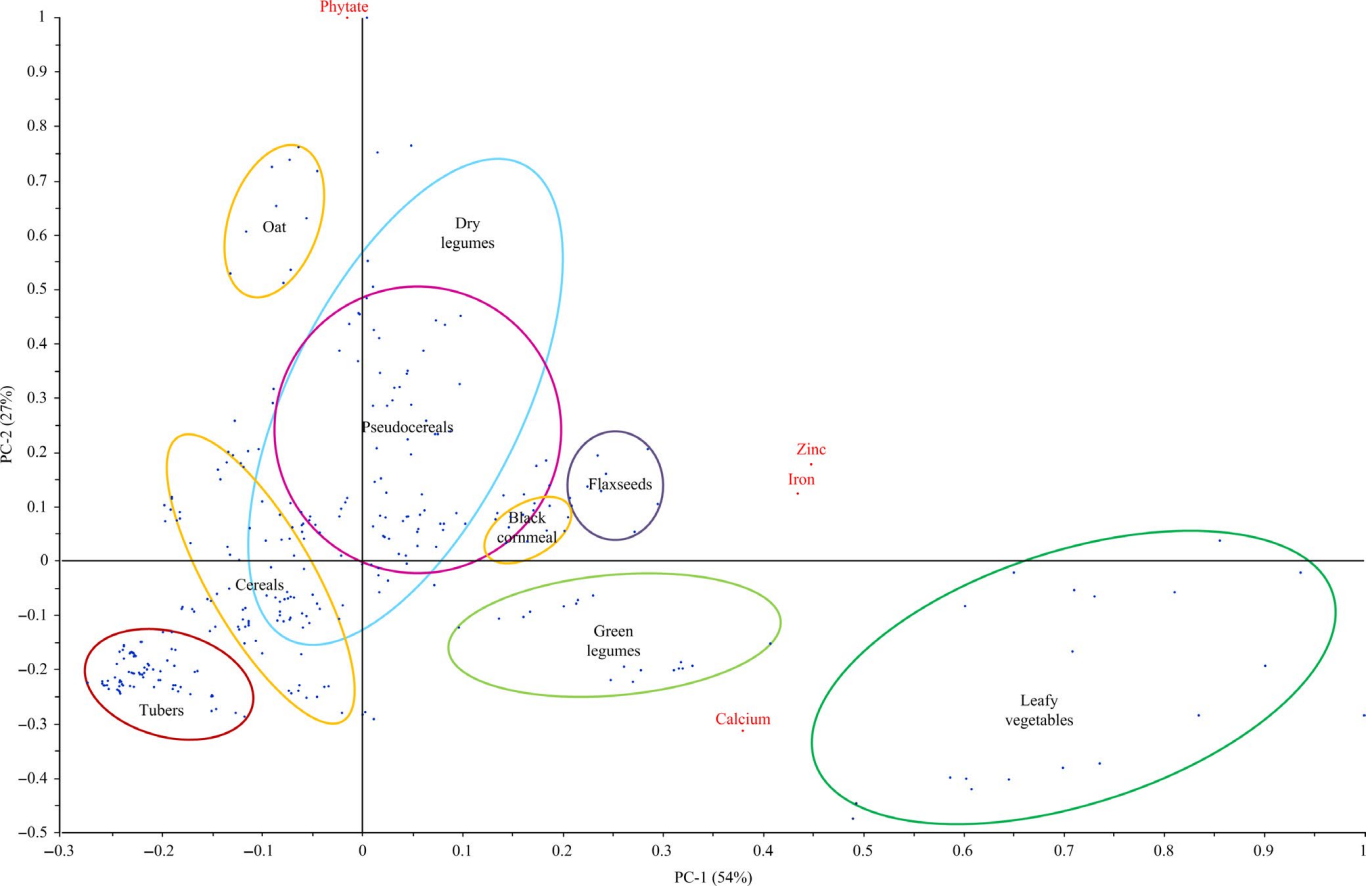
Principal component analysis biplot representing 33 common food components of diet from a rural area of Bolivia. The data set includes 17 food samples analyzed in the current study and 16 food samples analyzed by Lazarte, Carlsson, et al. (2015). The parameters included in the data set are iron, zinc, calcium, and phytate content expressed in dry matter

In order to have an overview of the nutritional implications of mineral and phytate content in diets of Chapare, Bolivia, and regions with similar situations, a PCA showing the relationship among food groups, minerals (iron, zinc, and calcium), and phytate content is presented in Figure 1. The PCA includes 33 common food components of the Chapare diet, and it was performed using The Unscrambler^®^ X 10.2 (CAMO software AS). Sixteen out of the 33 food samples were analyzed in our previous study (wheat grain, wheat flour, white bread, white maize, rice, noodles, quinoa, cassava roots, new cocoyam roots, potato imilla, potato runa, chuño, fava beans, lentils, peanuts, and plantain) (Lazarte, Carlsson, et al., 2015), and 17 samples are those presented in this study. The selected foods have shown to be the basis of the diet reported in Chapare, Bolivia, which covered more than 80% of the recommended nutrient intake of iron and zinc, but <40% of calcium requirements of rural population groups in Chapare (Lazarte, Alegre, Rojas, & Granfeldt, 2013; Lazarte, Soto, et al., 2015). Two principal components accounted for 81% of the variance in mineral and phytate content in the analyzed foods. The first principal component (PC1) explained 54% dataset variation and was positively loaded with zinc, iron, and calcium. The food groups leafy vegetables and green legumes are shown to be situated in this quadrant. The second principal component (PC2) explained 27% of dataset variation and was positively loaded with phytate content; the food groups pseudocereals, dry legumes and oats are also situated in this quadrant. Tubers have the lowest content of minerals and phytate (Figure 1). The Figure 1 shows that PCA which included previously analyzed foods provides an indication of relationships of food groups commonly consumed in Bolivia and their content of phytate and minerals.

## 5 CONCLUSION

The information provided here on the mineral and phytate content in 17 foods commonly consumed in rural areas of Bolivia is important to understanding the implications of a plant‐based diet for the nutritional status of this population. The best performing foods regarding mineral content are leafy vegetables and green legumes, as well as pseudocereals. However, an important factor to take into account with pseudocereals is their moderate to high phytate content, which is similar to some cereals and legumes. The estimated mineral bioavailability of the studied foods showed to be low due to molar ratios values were above the threshold, but Phy:Zn molar ratios for black cornmeal and yellow corn in cereals and dry peas in legumes showed a moderate estimated zinc bioavailability. Therefore, processing strategies and dietary diversification must be sought to reduce the phytate content in high‐mineral foods before consumption to ensure a good bioavailability in the human body.

## CONFLICT OF INTEREST

The authors declare no conflict of interest.

## ETHICAL STATEMENT

The authors declare that human and animal testing was unnecessary in this study.

## References

[fsn31127-bib-0001] Abd‐El‐Hady, E. A. , & Habiba, R. A. (2003). Effect of soaking and extrusion conditions on antinutrients and protein digestibility of legume seeds. LWT – Food Science and Technology, 36(3), 285–293. 10.1016/S0023-6438(02)00217-7

[fsn31127-bib-0002] Achidi, A. U. , Ajayi, O. A. , Maziya‐Dixon, B. , & Bokanga, M. (2008). The effect of processing on the nutrient content of cassava (*Manihot esculenta* Crantz) leaves. Journal of Food Processing and Preservation, 32(3), 486–502. 10.1111/j.1745-4549.2007.00165.x

[fsn31127-bib-0003] Alonso, R. , Aguirre, A. , & Marzo, F. (2000). Effects of extrusion and traditional processing methods on antinutrients and in vitro digestibility of protein and starch in faba and kidney beans. Food Chemistry, 68(2), 159–165. 10.1016/S0308-8146(99)00169-7

[fsn31127-bib-0004] AOAC (2000). Official methods of analysis of AOAC International. Gaithersburg, MD: AOAC International.

[fsn31127-bib-0005] Callegaro, M. , Milbradt, B. G. , Alves, E. , Diettrich, T. , Kemerich, D. M. , Hausen, B. S. , … Emanuelli, T. (2011). Effect of wheat bran and flaxseed on cadmium effects and retention in rats. Human & Experimental Toxicology, 30(8), 981–991. 10.1177/0960327110384526 20876162

[fsn31127-bib-0006] Carlsson, N. G. , Bergman, E. L. , Skoglund, E. , Hasselblad, K. , & Sandberg, A. S. (2001). Rapid analysis of inositol phosphates. Journal of Agricultural and Food Chemistry, 49(4), 1695–1701. 10.1021/jf000861r 11308312

[fsn31127-bib-0007] Chavez, A. L. , Bedoya, J. M. , Sánchez, T. , Iglesias, C. , Ceballos, H. , & Roca, W. (2000). Iron, carotene, and ascorbic acid in cassava roots and leaves. Food and Nutrition Bulletin, 21(4), 410–413. 10.1177/156482650002100413

[fsn31127-bib-0008] Dahdouh, S. , Grande, F. , Espinosa, S. N. , Vincent, A. , Gibson, R. , Bailey, K. , … Charrondière, U. R. (2019). Development of the FAO/INFOODS/IZINCG global food composition database for phytate. Journal of Food Composition and Analysis, 78, 42–48. 10.1016/j.jfca.2019.01.023 31057213 PMC6472536

[fsn31127-bib-0009] Desse, G. (2016). Comparison of three sweet potato (*Ipomoea batatas* (L.) Lam) varieties on vutritional and anti‐nutritional factors. Global Journal of Science Frontier Research, 16(4), 62–72.

[fsn31127-bib-0010] FAO (1992). Maize in human nutrition. Rome, Italy: Food and Agriculture Organization of the United Nations. 6086142

[fsn31127-bib-0011] FAO ( 2014). Food and nutrition in numbers 2014. Rome, Italy: Food and Agriculture Organization of the United Nations.

[fsn31127-bib-0012] Fordyce, E. J. , Forbes, R. M. , Robbins, K. R. , & Erdman, J. W. (1987). Phytate × Calcium/Zinc molar ratios: Are they predictive of zinc bioavailability? Journal of Food Science, 52(2), 440–444. 10.1111/j.1365-2621.1987.tb06634.x

[fsn31127-bib-0013] Fredlund, K. , Asp, N. G. , Larsson, M. , Marklinder, I. , & Sandberg, A. S. (1997). Phytate reduction in whole grains of wheat, rye, barley and oats after hydrothermal treatment. Journal of Cereal Science, 25(1), 83–91. 10.1006/jcrs.1996.0070

[fsn31127-bib-0014] Frontela, C. , García‐Alonso, F. J. , Ros, G. , & Martínez, C. (2008). Phytic acid and inositol phosphates in raw flours and infant cereals: The effect of processing. Journal of Food Composition and Analysis, 21(4), 343–350. 10.1016/j.jfca.2008.02.003

[fsn31127-bib-0015] Gibson, R. S. , Bailey, K. B. , Gibbs, M. , & Ferguson, E. L. (2010). A review of phytate, iron, zinc, and calcium concentrations in plant‐based complementary foods used in low‐income countries and implications for bioavailability. Food and Nutrition Bulletin, 31(2 Suppl), S134–S146. 10.1177/15648265100312S206 20715598

[fsn31127-bib-0016] Gibson, R. S. , & Ferguson, E. L. ( 1998). Food processing methods for improving the zinc content and bioavailability of home‐based and commercially available compllementary foods In USAID&FAO(Eds.), Micronutrient interactions: Impact on child health and nutrition (pp. 55–57). Washington, DC: International Life Science Institute Press.

[fsn31127-bib-0017] Harland, B. F. , & Morris, E. R. (1995). Phytate: A good or a bad food component? Nutrition Research, 15(5), 733–754. 10.1016/0271-5317(95)00040-P

[fsn31127-bib-0018] Haros, C. M. , & Schönlechner, R. ( 2017). Pseudocereals: Chemistry and technology. West Sussex: Wiley-Blackwell.

[fsn31127-bib-0019] Hídvégi, M. , & Lásztity, R. (2002). Phytic acid content of cereals and legumes and interaction with proteins. Periodica Polytechnica: Chemical Engineering, 46(1–2), 59–64.

[fsn31127-bib-0020] Hudson, R. , & Hanratty, D. (1991). Bolivia: A country study. Washington, DC: Federal Research Division, Library of Congress: For sale by the Supt. of Docs., U.S. G.P.O.

[fsn31127-bib-0021] Hurrell, R. , & Egli, I. (2010). Iron bioavailability and dietary reference values. The American Journal of Clinical Nutrition, 91(5), 1461S–1467S. 10.3945/ajcn.2010.28674F 20200263

[fsn31127-bib-0022] INE ( 2017). Encuesta de demografía y Salud EDSA 2016. Bolivia: Indicadores Priorizados La Paz: Instituto Nacional de Estadística.

[fsn31127-bib-0023] Khalil, A. H. , & Mansour, E. H. (1995). The effect of cooking, autoclaving and germination on the nutritional quality of faba beans. Food Chemistry, 54(2), 177–182. 10.1016/0308-8146(95)00024-D

[fsn31127-bib-0024] Khan, M. L. , Sharif, M. , Sarwar, M. , Sameea & Ameen, M. (2010). Chemical composition of different varieties of linseed. Pakistan Veterinary Journal, 30(2), 79–82.

[fsn31127-bib-0025] Konishi, Y. , Hirano, S. , Tsuboi, H. , & Wada, M. (2004). Distribution of minerals in quinoa (*Chenopodium quinoa* Willd.) seeds. Bioscience, Biotechnology, and Biochemistry, 68(1), 231–234. 10.1271/bbb.68.231 14745190

[fsn31127-bib-0026] Koplík, R. , Komínková, J. , Borková, M. , Mestek, O. , Kvasnicka, F. , & Suchanek, M. (2004). Effect of technological processing and maturity stage of seeds on the content and speciation of phosphorus and trace elements in peas. Food Chemistry, 87, 423–432. 10.1016/j.foodchem.2003.12.016

[fsn31127-bib-0027] Larsson, M. , & Sandberg, A. S. (1992). Phytate reduction in oats during malting. Journal of Food Science, 57(4), 994–997. 10.1111/j.1365-2621.1992.tb14340.x

[fsn31127-bib-0028] Latif, S. , & Müller, J. (2015). Potential of cassava leaves in human nutrition: A review. Trends in Food Science & Technology, 44(2), 147–158. 10.1016/j.tifs.2015.04.006

[fsn31127-bib-0029] Lazarte, C. E. , Alegre, C. , Rojas, E. , & Granfeldt, Y. (2013). Nutritional status of patients with cutaneous leishmaniasis from a tropical area of Bolivia, and implications for zinc bioavailability. Food and Nutrition Sciences, 4(10), 12 10.4236/fns.2013.410A009

[fsn31127-bib-0030] Lazarte, C. E. , Carlsson, N.‐G. , Almgren, A. , Sandberg, A.‐S. , & Granfeldt, Y. (2015). Original Research Article: Phytate, zinc, iron and calcium content of common Bolivian food, and implications for mineral bioavailability. Journal of Food Composition and Analysis, 39, 111–119. 10.1016/j.jfca.2014.11.015

[fsn31127-bib-0031] Lazarte, C. , Soto, A. , Alvarez, L. , Bergenståhl, B. , Medrano, N. , & Granfeldt, Y. (2015). Nutritional status of children with intestinal parasites from a tropical area of Bolivia, emphasis on zinc and iron status. Food and Nutrition Sciences, 6(4), 399–411. 10.4236/fns.2015.64041

[fsn31127-bib-0032] Lee, H. H. , Loh, S. P. , Bong, C. F. J. , Sarbini, S. R. , & Yiu, P. H. (2015). Impact of phytic acid on nutrient bioaccessibility and antioxidant properties of dehusked rice. Journal of Food Science and Technology, 52(12), 7806–7816. 10.1007/s13197-015-1918-9 26604353 PMC4648857

[fsn31127-bib-0033] Lestienne, I. , Icard‐Verniere, C. , Mouquet, C. , Picq, C. , & Treche, S. (2005). Effects of soaking whole cereal and legume seeds on iron, zinc and phytate contents. Food Chemistry, 89(3), 421–425. 10.1016/j.foodchem.2004.03.040

[fsn31127-bib-0034] Leterme, P. , Londono, A. M. , Estrada, F. , Souffrant, W. B. , & Buldgen, A. (2005). Chemical composition, nutritive value and voluntary intake of tropical tree foliage and cocoyam in pigs. Journal of the Science of Food and Agriculture, 85(10), 1725–1732. 10.1002/jsfa.2177

[fsn31127-bib-0035] Lönnerdal, B. (2002). Phytic acid–trace element (Zn, Cu, Mn) interactions. International Journal of Food Science & Technology, 37(7), 749–758. 10.1046/j.1365-2621.2002.00640.x

[fsn31127-bib-0036] Lopez, H. W. , Leenhardt, F. , Coudray, C. , & Remesy, C. (2002). Minerals and phytic acid interactions: Is it a real problem for human nutrition? International Journal of Food Science & Technology, 37(7), 727–739. 10.1046/j.1365-2621.2002.00618.x

[fsn31127-bib-0037] Luo, Y. , Gu, Z. , Han, Y. , & Chen, Z. (2009). The impact of processing on phytic acid, in vitro soluble iron and Phy/Fe molar ratio of faba bean (*Vicia faba* L.). Journal of the Science of Food and Agriculture, 89(5), 861–866. 10.1002/jsfa.3525

[fsn31127-bib-0038] Magallanes‐López, A. M. , Hernandez‐Espinosa, N. , Velu, G. , Posadas‐Romano, G. , Ordoñez‐Villegas, V. M. G. , Crossa, J. , … Guzmán, C. (2017). Variability in iron, zinc and phytic acid content in a worldwide collection of commercial durum wheat cultivars and the effect of reduced irrigation on these traits. Food Chemistry, 237, 499–505. 10.1016/j.foodchem.2017.05.110 28764025 PMC5544597

[fsn31127-bib-0039] Martínez, C. , Ros, G. , Periago, M. J. , Ortuño, J. , López, G. , & Rincón, F. (1998). In vitro protein digestibility and mineral availability of green beans (*Phaseolus vulgaris* L.) as influenced by variety and pod size. Journal of the Science of Food & Agriculture, 77(3), 414 10.1002/(SICI)1097-0010(199807)77:33.3.CO;2-4

[fsn31127-bib-0040] Masum Akond, A. S. M. G. , Crawford, H. , Berthold, J. , Talukder, Z. I. , & Hossain, K. (2011). Minerals (Zn, Fe, Ca and Mg) and antinutrient (Phytic acid) constituents in common bean. American Journal of Food Technology, 6(3), 235–243. 10.3923/ajft.2011.235.243 29861700 PMC5983041

[fsn31127-bib-0041] Mitchikpe, E. C. S. , Dossa, R. A. M. , Ategbo, E.‐A.‐D. , van Raaij, J. M. A. , Hulshof, P. J. M. , & Kok, F. J. (2008). The supply of bioavailable iron and zinc may be affected by phytate in Beninese children. Journal of Food Composition and Analysis, 21(1), 17–25. 10.1016/j.jfca.2007.06.006

[fsn31127-bib-0042] Morris, E. R. , & Hill, A. D. (1996). Inositol phosphate content of selected dry beans, peas, and lentils, raw and cooked. Journal of Food Composition and Analysis, 9(1), 2–12. 10.1006/jfca.1996.0002

[fsn31127-bib-0043] Nergiz, C. , & Gökgöz, E. (2007). Effects of traditional cooking methods on some antinutrients and in vitro protein digestibility of dry bean varieties (*Phaseolus vulgaris* L.) grown in Turkey. International Journal of Food Science & Technology, 42(7), 868–873. 10.1111/j.1365-2621.2006.01297.x

[fsn31127-bib-0044] Oberleas, D. , & Harland, B. F. (1981). Phytate content of foods: Effect on dietary zinc bioavailability. Journal of the American Dietetic Association, 79(4), 433–436. 7288050

[fsn31127-bib-0045] Periago, M. J. , Ros, G. , Martınez, M. C. , Rincón, F. , López, G. , Ortuño, J. , & Ros, F. (1996). In vitro estimation of protein and mineral availability in green peas as affected by antinutritive factors and maturity. LWT – Food Science and Technology, 29(5), 481–488. 10.1006/fstl.1996.0074

[fsn31127-bib-0046] Ratnayake, W. M. N. , Behrens, W. A. , Fischer, P. W. F. , L'Abbé, M. R. , Mongeau, R. , & Beare‐Rogers, J. L. (1992). Chemical and nutritional studies of flaxseed (variety Linott) in rats. The Journal of Nutritional Biochemistry, 3(5), 232–240. 10.1016/0955-2863(92)90045-K

[fsn31127-bib-0047] Reddy, N. R. , & Sathe, S. K. ( 2001). Food phytates. Boca Raton, FL: CRC Press.

[fsn31127-bib-0048] Repo‐Carrasco‐Valencia, R. , Acevedo de La Cruz, A. , Icochea Alvarez, J. C. , & Kallio, H. (2009). Chemical and functional characterization of kañiwa (*Chenopodium pallidicaule*) grain, extrudate and bran. Plant Foods for Human Nutrition, 64(2), 94–101. 10.1007/s11130-009-0109-0 19424801

[fsn31127-bib-0049] Repo‐Carrasco‐Valencia, R. A. , Encina, C. R. , Binaghi, M. J. , Greco, C. B. , & Ronayne de Ferrer, P. A. (2010). Effects of roasting and boiling of quinoa, kiwicha and kaniwa on composition and availability of minerals in vitro. Journal of the Science of Food and Agriculture, 90(12), 2068–2073. 10.1002/jsfa.4053 20582934

[fsn31127-bib-0050] Ruales, J. , & Nair, B. M. (1993). Saponins, phytic acid, tannins and protease inhibitors in quinoa (*Chenopodium quinoa*, Willd) seeds. Food Chemistry, 48(2), 137–143. 10.1016/0308-8146(93)90048-K

[fsn31127-bib-0051] Sandberg, A. S. , & Svanberg, U. (1991). Phytate hydrolysis by phytase in cereals: Effects on in vitro estimation of iron availability. Journal of Food Science, 56(5), 1330–1333. 10.1111/j.1365-2621.1991.tb04765.x

[fsn31127-bib-0052] Sanz‐Penella, J. M. , Wronkowska, M. , Soral‐Smietana, M. , & Haros, M. (2013). Effect of whole amaranth flour on bread properties and nutritive value. LWT – Food Science and Technology, 50, 679–685. 10.1016/j.lwt.2012.07.031

[fsn31127-bib-0053] Schlemmer, U. , Frolich, W. , Prieto, R. M. , & Grases, F. (2009). Phytate in foods and significance for humans: Food sources, intake, processing, bioavailability, protective role and analysis. Molecular Nutrition & Food Research, 53(Suppl 2), S330–S375. 10.1002/mnfr.200900099 19774556

[fsn31127-bib-0054] The World Bank Group (2019). Health nutrition and population statistics. Retrieved from https://databank.worldbank.org/data/source/health-nutrition-and-population-statistics#

[fsn31127-bib-0055] Umeta, M. , West, C. E. , & Fufa, H. (2005). Content of zinc, iron, calcium and their absorption inhibitors in foods commonly consumed in Ethiopia. Journal of Food Composition and Analysis, 18(8), 803–817. 10.1016/j.jfca.2004.09.008

[fsn31127-bib-0056] Umuhozariho, M. G. , Shayo, N. B. , Msuya, J. M. , & Sallah, P. Y. K. (2014). Cyanide and selected nutrients content of different preparations of leaves from three cassava species. African Journal of Food Science, 8(3), 122–129. 10.5897/AJFS2013.1100

[fsn31127-bib-0057] UNISDR ( 2012). Documento Pais Bolivia. VII Plan de Acción DIPECHO. Bogota, Colombia: UNISDR

[fsn31127-bib-0058] Valencia, S. , Svanberg, U. , Sandberg, A. S. , & Ruales, J. (1999). Processing of quinoa (*Chenopodium quinoa*, Willd): Effects on in vitro iron availability and phytate hydrolysis. International Journal of Food Sciences and Nutrition, 50(3), 203–211. 10.1080/096374899101247 10627836

[fsn31127-bib-0059] Villa, D. Y. G. , Russo, L. , Kerbab, K. , Landi, M. , & Rastrelli, L. (2014). Chemical and nutritional characterization of *Chenopodium pallidicaule* (canihua) and *Chenopodium quinoa* (quinoa) seeds. Emirates Journal of Food and Agriculture, 26(7), 609–615. 10.9755/ejfa.v26i7.18187

[fsn31127-bib-0060] Wang, N. , Hatcher, D. W. , & Gawalko, E. J. (2008). Effect of variety and processing on nutrients and certain anti‐nutrients in field peas (*Pisum sativum*). Food Chemistry, 111(1), 132–138. 10.1016/j.foodchem.2008.03.047

[fsn31127-bib-0061] Wang, N. , Hatcher, D. W. , Tyler, R. T. , Toews, R. , & Gawalko, E. J. (2010). Effect of cooking on the composition of beans (*Phaseolus vulgaris* L.) and chickpeas (*Cicer arietinum* L.). Food Research International, 43(2), 589–594. 10.1016/j.foodres.2009.07.012

[fsn31127-bib-0062] Wobeto, C. , Corrêa, A. , Abreu, C. , Santos, C. , & Abreu, J. (2006). Nutrients in the cassava (*Manihot esculenta* Crantz) leaf meal at three ages of the plant. Food Science and Technology (Campinas), 26(4), 865–869. 10.1590/S0101-20612006000400024

